# Discovery of the Lyme Disease Agent

**DOI:** 10.1128/mBio.02166-19

**Published:** 2019-09-17

**Authors:** Alan G. Barbour, Jorge L. Benach

**Affiliations:** a Department of Medicine, University of California Irvine, Irvine, California, USA; b Department of Microbiology & Molecular Genetics, University of California Irvine, Irvine, California, USA; c Department of Molecular Genetics & Microbiology, Stony Brook University, Stony Brook, New York, USA; d Department of Pathology, Stony Brook University, Stony Brook, New York, USA; Johns Hopkins Bloomberg School of Public Health

**Keywords:** Lyme disease, *Borrelia*, *Ixodes*, *Rickettsia*, *Babesia*, Stony Brook University, Rocky Mountain Laboratories, tick

## Abstract

A detailed first-hand account of the events leading up to the discovery of the Lyme disease agent has been lacking. Nearly 40 years have elapsed since the discovery of the organism that was named Borrelia burgdorferi. There are thousands of articles in the scientific and medical literature on this organism and the disease that it causes. In the interval since the organism’s discovery, however, misconceptions have arisen regarding not only the disease but the discovery itself. Accordingly, with this paper, we aim to fill in the details of this episode in medical history with a joint introduction, first-person accounts by the two authors, a summary of contemporaneous events, and concluding comments. The history of the discovery of the Lyme disease agent has threads originating in different places in the United States. Studies on Long Island, NY, provided the epidemiological thread of studies on rickettsial diseases and babesiosis, linking the latter with the cutaneous manifestation of Lyme disease, now known as erythema migrans. The Long Island thread intersected Montana’s Rocky Mountain Laboratories thread of studies on a relapsing fever *Borrelia* and its cultivation and expertise in vector biology. This intersection made possible the discovery of the spirochete and its recovery from patients. This paper stresses that what may seem to have been an individual scientific discovery is actually the product of several threads coming together and is attributable to more people than appreciated.

## Abstract (precis)

A detailed first-hand account of the events leading up to the discovery of the Lyme disease agent has been lacking. Nearly 40 years have elapsed since the discovery of the organism that was named Borrelia burgdorferi. There are thousands of articles in the scientific and medical literature on this organism and the disease that it causes. In the interval since the organism’s discovery, however, misconceptions have arisen regarding not only the disease but the discovery itself.

## PERSPECTIVE

The 18 June 1982 issue of the journal *Science* included an article entitled “Lyme Disease—a Tick-Borne Spirochetosis?” (1). Note the terminal question mark. The evidence linking the “treponema-like spirochete” isolated from ticks to an enigmatic seasonal illness in the northeastern United States was at that point circumstantial, namely, antibody reactivity to the microorganism in convalescent-phase sera from Lyme disease patients. However, within a year, the question mark could be dropped; the conjecture was proven in 1983. The “*Ixodes dammini* spirochete” was recovered from the blood and other specimens from patients with Lyme disease (2, 3), as well as from the rodents that are major hosts for the ticks (4, 5). By 1984, Lyme disease could legitimately be defined as a tick-borne bacterial zoonosis for which the main reservoirs in its life cycle were small animals; humans were inadvertent hosts.

Besides Stanley (Fred) Hayes, who performed the electron microscopy for the study in *Science* (1), we are the only surviving authors of that 1982 paper. Wilhelm (Willy) Burgdorfer (1925 to 2014), the article’s first author, subsequently recorded his recollections of the discovery in several short articles that appeared from 1984 to 1993 (6–11). Jonathan Edlow for his 2004 book, entitled *Bull’s-Eye: Unraveling the Medical Mystery of Lyme Disease*, drew from the available literature and several interviews to write a history of Lyme disease that was aimed at a general audience (12).

A detailed, documented, first-hand account of the events leading up to the discovery and the discovery itself has been lacking. We think that this is now warranted, not only because of the exigency of diminishing numbers of the original participants but also to correct misconceptions and inaccuracies in professional as well as popular-media versions that have grown into what amounts now to folklore.

Besides filling in the details of this episode in medical history, the following provides a more widely applicable, if not singular, lesson; what may seem an individual scientific discovery is actually the product of several threads coming together, attributable to more people than appreciated, and may owe as much to providence as it does to insight. The article is a combination of first-person accounts by each of us, our joint introduction, a summary of contemporaneous events elsewhere (Appendix), and concluding comments.

## FALL 1981 AND THE LEAD-UP

### Stony Brook, NY (J. L. Benach).

**FIG 1:**
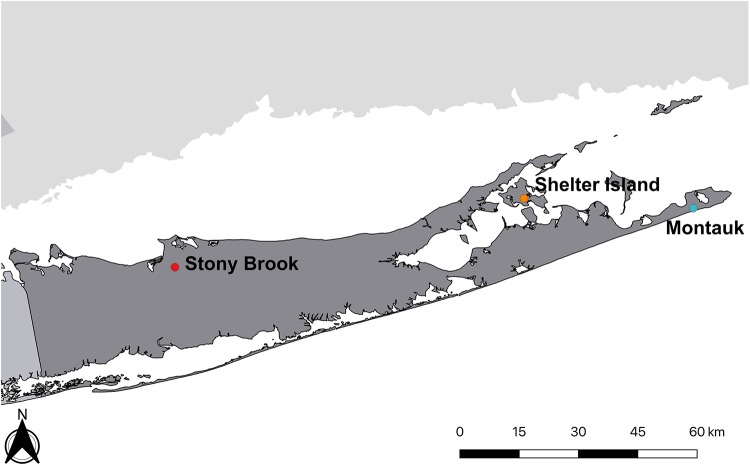
Map of Suffolk County, Long Island, NY, showing the locations mentioned in the narrative.

My graduate studies at Rutgers University in parasitology/entomology were on the development of filarial worms in mosquitoes. Classmates included Edward Bosler and Dennis White, who later join in this narrative about the discovery. After graduation, I joined the Bureau of Communicable Diseases of the New York State Department of Health in Albany, NY, in 1972 to work on recurring outbreaks of eastern equine encephalitis in Montauk, Long Island, NY (Fig. 1). The eastern part of Long Island includes four towns that are famous summer destinations for their historical interest and for their magnificent beaches. They also have a unique ecosystem that for over 30 or more years has seen large outbreaks of vector-borne diseases. In the early to mid-1970s, New York State and local health officials realized that there was a sharp increase in the populations of Dermacentor variabilis (American dog tick) across all of Suffolk County, the easternmost county of Long Island. Concomitant with the growing tick populations was an increase in the numbers of cases of Rocky Mountain spotted fever (RMSF) in the region, not only in the traditional easternmost towns but also in areas with higher population densities toward the western towns. There is historical evidence that an RMSF-like infection has been present on the eastern side of Long Island for decades.

**FIG 2:**
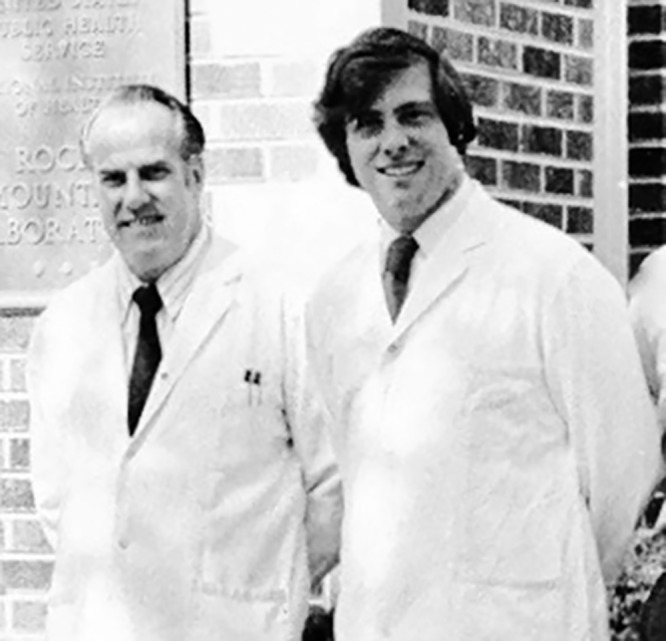
Jorge Benach and Willy Burgdorfer at Rocky Mountain Laboratories, Hamilton, MT, in June 1975.

Lacking the expertise for addressing the RMSF problem, I contacted Willy Burgdorfer at Rocky Mountain Laboratories (RML) of the National Institute of Allergy and Infectious Diseases (NIAID) in Hamilton, MT, to seek training in both the biology of the ticks and rickettsiology during 1974 to 1975 at RML (Fig. 2). After the training, I began my own studies of RMSF on Long Island. With the continued increase in the number of RMSF cases during 1971 to 1976, Dennis White, my Rutgers classmate, and I were transferred in 1976 from Albany to the Department of Pathology at the School of Medicine of Stony Brook University. We were to investigate this outbreak and to try to determine whether tick control was a feasible option to halt it. The results of these studies were published, jointly with Willy Burgdorfer (13). Nearly 150 cases of RMSF were documented from 1971 to 1976, including the deaths of several children and adults.

Screening for the presence of rickettsia in collections of D. variabilis was done by the hemolymph test (14). In this procedure, hemocytes extracted from the tick fluid are placed on a slide and stained for intracellular organisms. If rickettsia in a sample was suspected, additional hemocytes from the surviving tick could be tested further by indirect immunofluorescence with rickettsia-specific antisera. I learned this procedure from Willy and his RML colleague Robert Phillip, who also trained me in the serological assays for antibodies to the rickettsia. The level of infected *D. variabilis* ticks from Long Island was around 6% during the early 1970s, and infected ticks were tightly clustered in small geographic foci (15). The work related to RMSF and *D. variabilis* during those years was published in the monograph *Rickettsia and Rickettsial Diseases*, edited by Willy Burgdorfer and another RML scientist, Robert Anacker (16). During this same period, Barry Lissman, a veterinarian, and I reported the first clinical and laboratory description of canine RMSF (17).

Because we were limited in identifying the intrahemocytic organisms to the species level, numerous ticks were sent to Willy at RML for further identification. All of the rickettsias found in the *D. variabilis* ticks were identified as *Rickettsia montana* (now named Rickettsia montanensis sp. nov.). This was surprising, and we faced two options. The obvious one is that R. montanensis was the agent causing the human cases of RMSF, not the traditional agent, Rickettsia rickettsii. Although obvious, this was not a good option, as this species is thought to be a symbiont of *D. variabilis*. The second option was that another tick was transmitting the rickettsial agents, and this option led to the testing of human-biting *Ixodes* ticks on Long Island (see below).

Exchanges and collaborations between my lab at Stony Brook and the Burgdorfer lab at RML continued over this period and then lasted beyond the discovery of the Lyme disease agent. We continued to work with Willy with rickettsia as well as the Lyme disease spirochete up to his retirement in 1985. Throughout my period of training and thereafter, Willy was a caring mentor, and we developed a strong friendship. We had frequent visits with him and his wife, Dale, at their home in Hamilton, MT, and at meetings. During his visits to Stony Brook, he stayed at our home. For many years, Willy and I made it a point to talk on the telephone on our shared birthday.

### Role of babesiosis.

Babesia microti is a zoonotic hemoprotozoa (Apicomplexa) transmitted by ticks of the genus *Ixodes*. Concurrent with our rickettsia studies was a report in 1977 of the first case of human babesiosis on Shelter Island (Fig. 1) by Edgar Grunwaldt (1925 to 2014), the primary care physician serving the island (18). The same year, several cases of babesiosis on Nantucket Island were also reported (19). Thereafter, cases on both islands and on the “mainland” of Long Island accumulated rapidly, resulting in our several studies that established clinical and epidemiologic features of babesiosis (20–23).

The accumulating cases of babesiosis and the concurrent outbreak of RMSF created an alarming situation in the affected areas of Long Island. The evidence for transfusion-acquired babesiosis in premature infants and adults resulted in a ban imposed by the New York Blood Center on blood donations in the affected areas of Suffolk County. The Governor of New York, Hugh Carey, himself a summer resident of Shelter Island, became concerned with this problem, as did his Special Assistant for Health Affairs, Kevin Cahill. Cahill, a tropical medicine specialist in New York City, saw a number of babesiosis patients himself and took a scientific interest in this disease (24). He provided our lab with funds for increased studies on these tick-borne diseases. The support of Cahill to my lab extended to the work leading to the identification of the Lyme disease agent.

With additional funding, John McGovern and Peggy McGovern (nee Jacovina) joined my laboratory at Stony Brook to conduct studies on the isolation of B. microti in hamsters and then in mice, to develop a serological assay, and to map the reservoirs of this parasite (25, 26). Hamsters are the preferred laboratory animal to isolate and grow B. microti from ticks and from infected blood. That we did not recognize what may have been a concurrent spirochetal infection in hamsters may be due to the nonspecific manifestations of the spirochetal infection (27) and to the severe parasitemia in these animals, which overshadows the subtler signs of the borreliosis.

The blood donor collection ban anticipated the recognition that subclinical exposure may have been responsible for transmission cases and that these could be documented in a population of healthy individuals on Shelter Island. A 1978 serosurvey sought to determine the prevalence and incidence of asymptomatic and clinical babesiosis during a single transmission season. Mark Filstein, an Epidemic Intelligence Service (EIS) officer from the Center for Disease Control (CDC) and attached to the New York State Health Department, teamed with my laboratory at Stony Brook to set up the serosurvey and test the paired sera for antibodies to B. microti (28) from Shelter Island residents. In addition to detecting a point prevalence of 4.4% in the first serum collection in June and a 6.9% value in October, the serosurvey disclosed a number of *Babesia*-seroreactive individuals who reported a history of an erythema migrans. This serosurvey was pivotal in linking babesiosis with Lyme disease, which was being described in Connecticut at the same time. The sera used in the *Science* paper (1) came from the stored serum bank in my laboratory from the 1978 babesiosis Shelter Island survey (28). These sera were from survey volunteers who reported an erythema migrans and who were also seropositive for B. microti, and with one exception all of these patients had been previously diagnosed by Grunwaldt. The exception was a faculty member at Stony Brook University who, unknown to him at the time, had Lyme disease with all its known manifestations and kept a detailed account of his baffling illness. He was never treated and went through most of the known manifestations of Lyme disease. This patient’s serum was one of those examined for reactivity against the recently isolated spirochete from the Shelter Island ticks (see below and Alan Barbour’s account).

Additional searches for cases in 1979 and 1980 resulted in identifying several more persons both with antibodies to B. microti and with a history of erythema migrans (29). Edgar Grunwaldt, who was an astute diagnostician, continued to identify scores of patients with erythema migrans and Lyme arthritis, as well as patients with babesiosis. My lab worked closely with him and provided serological support and isolation attempts of B. microti in hamsters from Grunwaldt’s patients.

Having made the connection of erythema migrans (formerly called erythema chronicum migrans [ECM]) with babesiosis, many hypotheses about the etiology of the former were made. Through discussions with Willy, it was decided to start doing hemolymph tests on the *Ixodes* ticks beginning in the fall of 1981 that were collected around the homes of patients with erythema migrans or babesiosis on Shelter Island. Adult I. scapularis are most plentiful in the fall. Curiously, there were two cases of RMSF-like illness diagnosed in the fall of 1981 on Shelter Island (an unusual time of onset since cases of this disease are typically acquired in the spring to early summer), and this added another possibility to the increasingly more complex pattern of tick-borne diseases coexisting on Long Island at that time. Therefore, a shipment of adult *Ixodes dammini* (soon after reclassified as I. scapularis) ticks collected on Shelter Island in October 1981 were sent to RML. Willy never found any rickettsia in the hemocytes of I. scapularis ticks, nor were we aware then of the ubiquitous spotted fever group rickettsial ovarial symbiont of this tick, now known as *Rickettsia buchneri* (30). Instead and of great importance, examination of the hemolymph and midguts of these ticks revealed a filarial nematode (31) and spirochetes, observed by dark-field microscopy.

### Hamilton, MT (A. G. Barbour).

In the summer of 1980, our family moved north from Salt Lake City, UT, to the Bitterroot Valley of western Montana. I had just finished my clinical and research fellowship in infectious diseases (ID) at the University of Utah. John L. Swanson (1936 to 2013), an established investigator in bacterial pathogenesis, was my research mentor. John was recruited by Richard Krause, Director of the NIAID, to be chief of a new NIH laboratory at RML. After settling in, John asked me to join a group he was forming called the Laboratory of Microbial Structure and Function (LMSF). I accepted the offer of a position as senior staff fellow.

The personal thread of this story traces a bit further back, though, to a fourth-year medical school elective on the ID inpatient ward of Tuft University’s New England Medical Center. The division chief was Louis Weinstein, who attracted a series of outstanding ID fellows over the years. Two of these, Kenneth Ratzan and Martin Skinner, had been EIS officers of the CDC, a branch of the Public Health Service (PHS). Ken and Marty convinced me to apply to the EIS. Following a deferral from national service for 2 years of internal medicine residency, I entered the PHS as a commissioned officer and traveled to Atlanta for the CDC’s legendary crash course in epidemiology and statistics. My assignment was the Utah State Department of Health, where I served 2 years as an epidemiologist.

During 6 years in Utah, first as an EIS officer and then as a chief medical resident and ID fellow, I had experiences in the field and in hospitals with several arthropod-borne zoonoses, including plague, tularemia, Colorado tick fever, and RMSF but not Lyme disease. In the late 1970s, Lyme disease was beginning to take off in the northeastern United States, as Jorge relates above, but not anywhere close to its level in Utah. The prevailing expert opinion in the United States was that Lyme disease was most likely caused by a virus and was self-limiting (32). The article in the CDC’s *Morbidity and Mortality Weekly Report* reporting on the probable effectiveness of antibiotics for treatment was not until May 1980 (33).

In late 1979 and early 1980, my research attention was instead on strains of Staphylococcus aureus that caused tampon-associated toxic shock syndrome, which was especially frequent in Utah in that epidemic (34). I had earlier embarked on a study of beta-lactam antibiotic action on Neisseria gonorrhoeae (35). These were the research topics on my mind when we arrived in Hamilton, MT. On board at RML as of July 1980 and with laboratory space and the technical assistance of Sandra Tessier, I continued my studies of S. aureus pathogenesis (36) and of beta-lactam antibiotic action (37) during the remainder of that year and the first part of 1981.

RML was one of the first dedicated laboratory facilities of the PHS (https://www.niaid.nih.gov/about/rocky-mountain-history). Its history begins with Henry Ricketts’ studies of RMSF, which was a threat to the growing population of western Montana in the early 20th century. This heritage established RML’s preeminence within the NIH for research on tick-borne diseases. As Jorge tells, Willy Burgdorfer was one senior investigator in the field of ticks and tick-borne diseases at RML.

Willy came to RML in 1950 after completing his Ph.D. in medical entomology in Switzerland (38). His thesis was on the interactions of the African relapsing fever agent, Borrelia duttonii, with its tick vector, *Ornithodoros moubata* (39). While relapsing fever never ceased to be in RML’s portfolio, in the 1950s through the 1970s, it was considered a lower priority than rickettsial diseases as a public health threat. By Willy’s account, he was encouraged by his supervisors to switch his focus from the spirochetes of relapsing fever to rickettsial diseases (38).

Willy was a neighbor; he and Dale lived across the street from us. Willy was also the occasional play-by-play announcer for the youth soccer matches that we attended, but at work, his lab was in a distant wing on another floor of the building, and he was affiliated with the Epidemiology Branch, a different unit at RML, so I was not familiar with his research.

More convenient, just down the hall, was the lab of Herbert Stoenner. Herb had been the administrator of RML when it comprised a single NIAID laboratory. After RML’s reorganization into separate laboratories for bacteria and for viruses as well as the Epidemiology Branch, Herb retained an independence from the other units at RML. Over his career, Herb had mainly studied host aspects of various zoonoses. For several years, his research interest had been the relapsing fever agent Borrelia hermsii and the phenomenon of antigenic variation that it manifested. I learned of Herb’s research on relapsing fever and recognized possible analogies with the antigenic variation of N. gonorrhoeae that John Swanson was studying in his lab. However, first Herb and I collaborated on a study of the action of penicillin on B. hermsii and a characterization of the penicillin-binding proteins of that organism with a technique that I had used for N. gonorrhoeae (40). In the early 1970s, Herb had introduced to RML Richard Kelly’s breakthrough medium for continuously propagating relapsing fever spirochetes in broth culture (41).

Herb’s and my next collaboration was based on antigenic variation during relapsing fever. Over several years, Herb had developed a remarkable set of isogenic serotypes of B. hermsii and serotype-specific antisera to each of them (42). This culminated in the identification of the variable antigens of B. hermsii (43). This discovery subsequently led to a series of studies on the genetics of the antigenic variation of the agent of relapsing fever.

In early 1981, I implemented in my lab Herb’s method for growing relapsing fever spirochetes. In the interval since his paper on the growth characteristics of B. hermsii in Kelly’s formulation, Herb had modified (or “fortified” in his words) the formula with additional ingredients. Unlike the original Kelly’s medium, which required hundreds of cells to establish growth and which did not sustain viability in the stationary phase (44), what we called “modified Kelly’s medium” (MKM) allowed the growth of B. hermsii cultures from single-cell inocula and longer maintenance of the viability of cells in stationary phase. The description of this formulation and its improved performance was not published until 1982 (40, 42).

The abrupt change in focus of my research to Borrelia hermsii was supported and encouraged by John Swanson. By job description, I was in a postdoctoral position in LMSF, and John could have required that I continue to work on N. gonorrhoeae, the subject of his research, but from the time I had walked into his office as an ID fellow to ask about antibiotic resistance and he had handed me a book on bacterial genetics, John had granted me considerable freedom, but he was not aloof. He was generous of his time for Socratic-style mentorship.

Until the late 1981 events described here, there was no research program dedicated to Lyme disease at RML, although the association of Lyme disease with ticks had been established more than 2 years before (45), and RML was the go-to tick-borne disease lab for the NIH.

**FIG 3:**
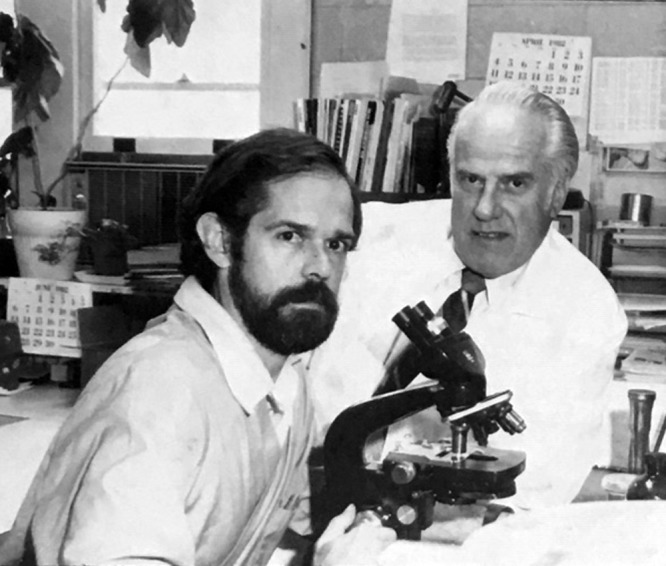
Alan Barbour and Willy Burgdorfer at Rocky Mountain Laboratories, Hamilton, MT, in June 1982. (Photo courtesy of Rocky Mountain Laboratories.)

Jorge’s collection of adult *I. dammini* ticks from Shelter Island was sent to Willy in October 1981. I was in Chicago from 4 to 6 November 1981 attending the Interscience Conference on Antimicrobial Agents and Chemotherapy. I heard Allen Steere’s plenary talk on Lyme disease and his conclusion from their controlled trials that it was treatable with penicillin. This was the first that I paid attention to Lyme disease, but given my recent studies of penicillin, what struck me was the antibiotic susceptibility of the still-unknown agent. When I returned to Hamilton, I stopped by Willy’s office, told him about my recent trip, and asked him what he knew about Lyme disease (Fig. 3). By Willy’s 1993 account, I told him that Steere had talked about a leptospire that had been isolated (11), but that is not my recollection. My first awareness of the connection of a leptospire with Lyme disease was weeks later, as described in the Appendix. Allen Steere confirmed that he would not have presented on the isolation of a leptospire at that conference because it was a CDC finding (A. C. Steere, personal communication).

Whatever the case, Willy told me about his recent observation of spirochetes in the midguts of *I. dammini* ticks that he had received from Jorge and had been examining in search of rickettsias. We agreed that I would try to isolate the organism from the ticks. On 13 November 1981, Willy provided me with tick midgut contents broken up and suspended in buffer (46). The ticks had been dipped in ethanol and then briefly flamed to sterilize their exterior before dissection. I made 10-fold dilutions of the suspension and inoculated tubes of MKM medium. We reported on the eventual pure culture obtained from the 10^−3^ dilution (1), but my first observation of motile spirochetes in the medium was of the 10^−1^ dilution on the 17th of November. However, this culture had another bacterium growing in it: a motile Gram-negative rod. I spent several days trying to clear what we presumed to be a contaminant with different antibiotics. I eventually found that nalidixic acid and 5-fluorouracil (which had been used in selective media for another spirochete, Leptospira interrogans) could suppress if not eliminate the other organism while allowing growth of the spirochete.

While we could not make a serious claim with a contaminated culture, it was the first recovery of the spirochete outside the tick and the only one with which to work at the time. Sandy Tessier and I injected it into different strains of mice, including T-cell-deficient nude mice, but we were accustomed to monitoring the densely spirochetemic B. hermsii infections by microscopy of wet mounts of blood. If there had been spirochetes in the blood of any of the animals, they would have been too low in concentration for detection by microscopy (47). None of the mice, including the immunodeficient animals, became discernibly ill or otherwise disabled.

I also used cell suspensions, which were mostly the spirochete but still contained the Gram-negative rod, on 30 December 1981 for indirect immunofluorescence assays (IFA) with some of the sera provided by Jorge and observed fluorescence several dilutions out with patient sera but not control sera. By Western blotting of a culture lysate, we observed several bands in the *Ixodes dammini* spirochete (IDS) lane of the convalescent-phase sera of a Lyme disease patient but none with convalescent-phase sera from someone who had toxic shock syndrome in Salt Lake City, UT. There was also specificity of the Lyme disease sera for the IDS. There were fewer bands in another lane containing B. hermsii. Again, these were not results that could be included in a paper, but they gave us confidence that we were onto something.

Willy at the same time was performing an immunofluorescence assay (IFA) using these sera on infected midgut preparations (11). These were with sera from Edgar Grunwaldt’s practice and an additional serum that Jorge had stored in his lab from the 1978 serosurvey. These were the results that are given in the 1982 paper (1).

The population of spirochetes that eventually grew out of the 10^−3^ dilution free of contamination was subsequently cloned by limiting dilution. The lab name for this clonal population was “B31,” which signified the first isolate of the 3 B’s of Burgdorfer, Benach, and Barbour. This stuck as the official name for the type strain of the species. We recently determined that the original uncloned isolate from the midguts of a set of ticks was (unsurprisingly given the high prevalence of infection in ticks and the strain diversity of B. burgdorferi) a mixture of two different strains of B. burgdorferi. What was designated B31 was the more numerous of the two, thus its selection in pure culture by serial dilution. However, there was also a strain of the OspC N genotype that was recovered from the blood of a mouse that had been inoculated with the uncloned population (48).

During this period, Willy carried out experimental infections of a few rabbits using other ticks from Shelter Island that had been provided by Jorge (1). These resulted in infections of the rabbits, which we confirmed by Western blot analyses of antibodies against lysates of the pure cultures.

## THE YEAR AFTER THE DISCOVERY

### Stony Brook, NY (J. L. Benach).

James Coleman joined my lab in 1979 and became an integral part in the discovery of the Lyme disease agent in subsequent studies. Jim Coleman and I traveled to RML in the spring of 1982 to work with Alan Barbour on cultivation of the newly discovered organism. Immediately following this visit, my lab began attempts to culture the organism from the patients during the 1982 tick season. A network of physicians in the areas of endemicity that had been assembled for the earlier studies on babesiosis were asked to submit blood specimens for culture. Two isolates were derived from 16 blood specimens in the first 2 months (2). The ratio of positive cultures in blood to total blood samples tested in that year is similar to the current ratio, but at that time, we were not aware that the period of spirochetemia in Lyme disease is short and not useful for the culture of this organism and, hence, for diagnosis. The isolation of the spirochetes from patients fulfilled Koch’s postulates. This effort was supported with funds from Robert Edelman of the Clinical and Epidemiological Studies Branch of the NIAID in Bethesda, MD, and Edelman was also a coauthor of the 1983 *New England Journal of Medicine* paper (2). James Coleman was an essential participant in all of the studies before the discovery of the Lyme disease agent, leading up to it, and thereafter. Jim became a gifted spirochetologist with unmatched cultivation skills. At this time, John Hanrahan, another EIS officer stationed at the New York State Department of Health, joined the activities of the Benach lab. His input was critical in all the epidemiological considerations that were required for all the studies during this period and thereafter, including the isolation of the organism from patients. Additional epidemiological studies were conducted on Long Island by John Hanrahan and the Benach lab documenting the increasing presence of Lyme disease on Long Island (49, 50).

Edward Bosler (another Rutgers classmate) joined the Stony Brook lab in 1981. He conducted extensive field studies in the areas of endemicity and tested a number of mammals. His studies pointed clearly to Peromyscus leucopus, the white-footed mouse, as the main reservoir of the Lyme disease spirochete (5). The ability to culture the *Ixodes dammini* spirochete reliably from wildlife as well as from ticks was key to deciphering the natural history of this pathogen, and thus, Bosler extended the original reservoir identification (51). Culturing the spirochete also proved to be critical in extending the range of the disease spectrum.

Barry Lissman, the veterinarian who described canine RMSF 4 years earlier, now was able to describe canine Lyme disease for the first time. His clinical observations of lameness in dogs infested with the *Ixodes* ticks matched the positive serology as well as the spirochete isolations made at the Benach lab from Lissman’s dogs (52). The burgeoning field of canine Lyme disease had its beginning in this collaboration, which dates back to the RMSF studies 4 years earlier.

During this entire period of discovery, close collaborations between RML and the Stony Brook lab continued. As a fitting end to the loop, Grunwaldt, Barbour, and Benach documented concurrent Lyme disease and babesiosis in a patient with an iron-clad laboratory diagnosis of both diseases (53).

### Hamilton, MT (A. G. Barbour).

After the discovery but before publication of the paper, Willy and I heard that the CDC had isolated a spirochete from a Lyme disease patient. We sent Fred Hayes’ electron photomicrographs of the *Ixodes dammini* spirochete to George Schmid and his colleagues at the CDC, and they sent us corresponding pictures of their spirochete. We let out our collective breath when we saw in their photographs a typical leptospire. This had been isolated from a skin specimen of one individual but not subsequently from any other patients. Schmid et al. later described what turned out to be a new type of *Leptospira*, but it was not the cause of Lyme disease (54).

Some of the other activities at RML during the immediate postdiscovery phase included further improvement in the culture medium for the organism (46), collaborations with Stony Brook University and Yale University in isolating and characterizing the IDS from patients with Lyme disease (2, 3), identification of a closely related spirochete in Ixodes ricinus ticks in Switzerland (55, 56), further studies of the rabbit model of the infection (7), a collaboration with Edgar Grunwaldt and Allen Steere that led to the identification of several of the major immunogenic proteins of the organism (57), and the beginning of many characterizations in our or other laboratories, including Stony Brook’s, of novel proteins of these bacteria (in the first case, the OspA protein of the outer membrane) (58).

### Concluding comments (J. L. Benach and A. G. Barbour).

In fall 1983, the inaugural international conference on Lyme disease was held in New Haven, CT, and organized by Allen Steere, Steven Malawista, and their colleagues at Yale University. This is an appropriate event to end this narrative, about 2 years after the discovery. The meeting was small enough to be accommodated by a medium-sized lecture hall of the college. The proceedings and papers from that meeting are publicly accessible in the archives of the *Yale Journal of Biology and Medicine* (https://medicine.yale.edu/yjbm/). At the conference, Fred Hyde and Russell Johnson of the University of Minnesota (59) and George Schmid of the CDC and several coauthors (60) presented DNA-DNA hybridization, morphologic, and other phenotypic evidence that this newly discovered spirochete was in a cluster with the *Borrelia* species that cause relapsing fever and was neither a treponeme nor a leptospire. Near the end of the conference, there was a discussion among attendees in the audience about what to name the new *Borrelia* species. Klaus Weber from Germany nominated “*burgdorferi*.” Other suggestions recognized either a feature of the disease, e.g., “*rheumatica*,” or a relevant place, e.g., “*lymei*.” A vote was taken, and “*burgdorferi*” carried. This was subsequently incorporated into the formal description of the species by Johnson et al. the next year (61).

The accompanying Appendix reviews other attempts to identify the cause of Lyme disease or ECM in North America and Europe after 1975. Much of it is a story of near misses and unrecognized clues but not confined to a single laboratory. Rewind the clock and start again under somewhat different circumstances, and others might be writing this history piece instead. We acknowledge the several contingencies along the way, but this probably could also be said of other discoveries in biology and medicine. Our motivation was not only to record the events in both a more detailed and personal way but also and more importantly to recognize the many other people that made this discovery possible, whether they were aware of that or not.

These discoveries were also achieved through the collaboration of many agencies and institutions, specifically, Rocky Mountain Laboratories of the National Institute of Allergy and Infectious Diseases, National Institutes of Health, Stony Brook University, the New York State Department of Health, the Centers for Disease Control and Prevention, the Suffolk County Department of Health, and private community physicians and veterinarians. Thirty-seven years after the *Science* paper (1), there are over 30,000 studies in PubMed listed under the headings “Borrelia” and “Lyme disease” from scientists from all over the world.

## APPENDIX

### OTHER ATTEMPTS TO FIND THE CAUSE OF LYME DISEASE

What follows is a summary of prior and contemporaneous events that are relevant to the search for the cause. These are not from our first-hand experiences but assembled from the literature and from personal communications with those involved, either at the time or through more-recent contact. We do not claim comprehensiveness. Jonathan Edlow in his book on the history of Lyme disease also provides information on the activities at other institutions (12). The point is that there were several other individuals and research groups who were close to identifying the cause but for one reason or another did not succeed. In other cases, the significance of what in retrospect was observation of the etiologic agent was not apparent at the time.

In the 19 July 1976 issue of the *Journal of the American Medical Association*, one of the Medical News articles featured this heading: “What May Be a New Form of Arthritis Is Discovered in New England Area” (62). The story reported on a presentation by Allen Steere at that year’s American Rheumatism Association meeting and on follow-up interviews. According to the news article, the similarity of the skin lesion that patients reported to a “cutaneous skin lesion first reported in Europe more than 50 years ago [previously] and believed to result from a tick bite” had been noted. The article continued that the investigators “strongly suspect[ed] an infectious agent,” but that “so far, all cultures and serological tests have failed to point to a specific agent.” The investigators were “particularly interested in testing the patients for the group A arthropod-borne viruses that cause joint diseases.” The full journal article by Steere et al. of the investigation elaborates on the cases and the search for the cause (63). The latter included cultivation of synovium and joint fluid for various bacteria using standard media of clinical microbiology laboratories for aerobic and anaerobic bacteria, mycoplasmas, and viruses. The serological assays measured antibodies to various rickettsias, *Coxiella*, leptospires, and mycoplasmas as well as viruses, but these were unrevealing.

In other issues of *JAMA* of that year, Mast and Burrows reported in two letters their experiences as physicians at the U.S. Navy medical center in Groton, CT, with what they recognized as erythema chronicum migrans (ECM) as well as their anecdotal findings on treatment (64, 65): “all erythemas responded dramatically to treatment with penicillin or erythromycin.” While they concluded that the ECM was “infectious,” “antibiotic-sensitive,” and “probably transmitted by an arthropod vector,” Mast and Burrows also inexplicably described it as “nonbacterial.” Rudolph Scrimenti in a case report described a patient with ECM and an apparent response to penicillin (66), but the relationship of this case in Wisconsin to the illnesses in the northeastern United States was overlooked at the time.

In West Germany during this period of the late 1970s, two clinicians independently searched for a cause for ECM and related disorders. These were Klaus Weber in Munich and Rudolf Ackermann in Cologne. Weber suspected a bacterial cause (67) and sent skin biopsy specimens to the microbiology institute in Munich, Germany, where the specimens were cultured for bacteria but not in a medium suitable for borrelia (12). Ackerman also suspected an infectious cause and demonstrated in the sera of ECM patients antibodies against the relapsing fever agent Borrelia duttonii, which is cross-reactive with B. burgdorferi (68), but did not isolate a spirochete.

Edgar Grunwaldt of J. L. Benach’s narrative had been aware of erythema chronicum migrans in Europe and in his Shelter Island medical practice had been diagnosing this and treating it with antibiotics after reading the literature but then stopped in 1978 when the prevailing expert opinion was that the disorder was self-limited (69).

By 1979 and 1980, investigators at Yale were beginning to suspect that Lyme disease and ECM were caused by a bacterium (A. C. Steere, personal communication). Allen Steere had been in communication with Willy Burgdorfer and had sent him several coded sera. These were tested at RML for antibodies against several *Rickettsia* spp., including what Willy called the “Swiss agent.” This organism had been isolated from Ixodes ricinus ticks and subsequently designated the species Rickettsia helvetica (70, 71). Our review of the results that are contained in correspondence between Willy and Allen Steere in 1980 (archived at https://uvu.contentdm.oclc.org/digital/collection/Burgdorfer) did not find evidence of a specific antibody response to a *Rickettsia* sp., including the “Swiss agent,” in the course of Lyme disease infection.

The attempt that perhaps came closest to identifying the cause was at the CDC. According to George Schmid’s account that was recorded in Edlow’s book, CDC investigators tried to isolate an agent in Kelly’s medium, as well as in a variety of other media and experimental animals (12). However, this would have been the early version of the medium that would not likely have supported growth from the low inoculum found in ticks (44). The modified Kelly’s medium (42), which we showed did support the growth of B. burgdorferi from ticks, was unknown outside RML at the time.

There were also at least three independent occasions in 1980 or earlier when a researcher had observed spirochetes in ticks during the period when a viral cause was uppermost in most peoples’ minds. Joseph Piesman, when he was at Harvard, had noted spirochetes in some Nantucket Island I. scapularis ticks that he was examining for Babesia microti but consulted with colleagues and mistakenly concluded that the spirochetes were probably symbionts of the ticks (J. Piesman, personal communication).

At Yale, William Krinsky, who had trained with Willy Burgdorfer, had noted spirochetes in *Ixodes dammini* ticks from Connecticut in 1978 and had tested them with what was thought to be convalescent-phase serum from a Lyme disease patient. There was no fluorescence. Later he found out that the serum had in fact no antibodies to B. burgdorferi (W. L. Krinsky, personal communication).

Charles (Ben) Beard remembers that Wilbur Downs, his former mentor and arbovirus expert at Yale, had told Ben before he died in 1991 that he had injected chick embryos with tick material in the search for a virus, but to his later chagrin interpreted the spirochetes that grew in the chick embryos as “contaminants” (C. B. Beard, personal communication).

It is possible that in one or more of these cases, the observed spirochetes were actually Borrelia miyamotoi and not B. burgdorferi. The presence of B. miyamotoi in I. scapularis ticks, which vector B. burgdorferi, was not recognized until 2001 (72). In that alternate scenario, an attempt to cultivate the spirochete in the improved medium may in the end have come to naught because of the great difficulty of cultivating B. miyamotoi, especially directly from ticks.
